# Supplementary material for: Estimating personal temporal symptom networks for childhood cancer survivors
Source: Commun Med (Lond). 2025 Aug 29;5:377. doi: 10.1038/s43856-025-01105-0 (PMC12394638; doi:10.1038/s43856-025-01105-0)
Supplement: Supplementary file 2 — Supplementary Information [file 43856_2025_1105_MOESM2_ESM.pdf]

Supplementary Note I: Diagnostic Testing of Model Assumptions

An essential aspect of inference for the fitted autoregressive logistic regressions with covariates is assessing potential violations of statistical assumptions. Therefore, for the real-data application, we performed the following diagnostic tests to evaluate these assumptions.

1) **Independence of errors/observations:** Since the autoregressive logistic model estimates correlations between symptoms observed at the previous and current timepoints, it is essential to assess whether the residuals from the time series model exhibit autocorrelation (i.e., whether they are independent over time). To evaluate this, we applied the Ljung-Box test, where the null hypothesis states that the residuals are independently distributed (i.e., no autocorrelation), and the alternative hypothesis indicates the presence of autocorrelation. In our real-data application, we conducted 100 iterations of data splitting inference. For each iteration, we performed the Ljung-Box test on the residuals for each of the ten symptom domains and computed the mean p-value across all iterations. The summarized p-values, presented in Supplementary Table 1, indicate that the null hypothesis was not rejected, suggesting no evidence of autocorrelation in the residuals.

2) **Absence of multicollinearity:** Assessing the absence of multicollinearity in autoregressive logistic regression models with LASSO can be challenging because LASSO inherently performs variable selection and shrinks correlated predictors toward zero, thereby reducing concerns about multicollinearity. However, some degree of multicollinearity may still exist among the selected predictors. To evaluate this, we calculated the Variance Inflation Factor (VIF) for the variables retained by LASSO in the fitted autoregressive logistic regression models. VIF quantifies the extent to which the variance of a regression coefficient is inflated due to multicollinearity among predictor variables, helping to assess whether independent variables are highly correlated. Similar to the Ljung-Box test, we computed the mean VIF for the selected variables in the regression models of each of the ten symptom domains across 100 iterations of data-splitting inference. The averaged VIF values, presented in Supplementary Table 2, are all below 5, suggesting that multicollinearity is not a concern in our autoregressive logistic regression models.

3) **Fixed predictors:** In our autoregressive logistic regression model, fixed predictors are those that remain constant over time. These include individual characteristics (i.e., age at the baseline survey, sex, race/ethnicity, attained education, annual personal income, marital status, and ever receiving chemotherapy and radiation therapy). Assessing fixed predictors in an autoregressive logistic regression model with LASSO typically involves examining their consistency in selection across different models and evaluating their impact on the outcome. Since we performed 100 iterations of data-splitting inference and used bootstrapping to construct 95% confidence intervals (CIs) for the estimated coefficients, our data analysis procedure inherently assessed the role of fixed predictors in the fitted models. The identified individual characteristics that significantly influence the correlations between symptoms highlight the importance of fixed predictors in understanding symptom dynamics over time.

4) **Outlier:** Identifying outliers in an autoregressive logistic regression model with LASSO requires examining influential observations that may disproportionately affect model estimates. In our real-data application, we employed standardized residuals to detect potential outliers in the fitted autoregressive logistic regression model. Specifically, we computed the standardized deviance residuals and identified observations with absolute values greater than 3, as these may indicate potential outliers. Consistent with the Ljung-Box test performed above, we calculated the residuals for fitted models across each of the ten symptom domains over 100 iterations of data-splitting inference. The average number of observations with absolute standardized residuals greater than 3, as presented in Supplementary Table 3, is zero or nearly zero, suggesting that outliers are not a concern in our analysis.

5) **Correctly specified models and correct functional forms:** To evaluate whether the autoregressive logistic model is correctly specified and has the appropriate functional form, we performed the Hosmer-Lemeshow (HL) goodness-of-fit test, which assesses how well the predicted probabilities align with observed outcomes by dividing the data into deciles of risk and comparing expected versus observed event rates. The null hypothesis states that the model is correctly specified, meaning there is no significant difference between observed and predicted probabilities, while the alternative hypothesis suggests model misspecification due to a mismatch between observed and predicted values. Similar to

the Ljung-Box test, we applied the HL test to each of the ten symptom domains and computed the mean p-value across 100 iterations of data-splitting inference. The summarized p-values, presented in Supplementary Table 4, indicate that the null hypothesis was rejected, suggesting evidence of model misspecification. This result is not surprising, given the strong assumptions made in modeling symptom correlations over time using the autoregressive logistic model. Several factors may contribute to model misspecification. First, the model only includes a limited set of symptom domains for temporal network construction, and the omission of other relevant symptoms may lead to misspecification. Second, the model assumes that symptoms at the current timepoint depend only on symptoms at the immediately preceding timepoint (lag-1 factorization), whereas symptoms from two or more prior timepoints may also influence current symptoms, a limitation of the current dataset. Third, logistic regression assumes a linear relationship between predictors and the log-odds of the outcome, which is a strong assumption that may not hold in reality; nonlinear relationships may better capture symptom interactions, and alternative approaches such as generalized additive models or machine learning algorithms may provide a more flexible framework. Fourth, due to the model's complexity, we included only a few individual characteristics such as basic demographics and socioeconomic status, while a more comprehensive set of patient-specific factors plus neighborhood-level social determinants of health could improve model specification. Therefore, future evaluation is warranted by incorporating additional symptom domains to reduce omitted variable bias, extending the autoregressive structure to include longer lag terms or more timepoints, exploring more flexible modeling approaches such as generalized additive models or machine learning algorithms to capture nonlinear dependencies, and expanding individual- and neighborhood-level covariates to enhance predictive accuracy and robustness. While there is evidence of model misspecification, as far as we are aware, this study is the first to explore the estimation of personal temporal symptom networks using an autoregressive logistic framework. Our primary goal is not to determine whether the autoregressive logistic model is perfectly specified, but rather to expand this field by gaining insight into how symptoms evolve and correlate over time. Despite this limitation, our findings provide valuable insight to understanding temporal symptom dynamics and lay the foundation for future methodological advancements in this area.

6) **Pseudo R-squared:** Pseudo R-squared is another measure of goodness-of-fit for autoregressive logistic regression models with LASSO. Here, we report the results of McFadden's R-squared. Consistent with the tests performed above, we calculated McFadden's R-squared for the fitted autoregressive logistic regression models across each of the ten symptom domains over 100 iterations of data-splitting inference. The average McFadden's R-squared values, presented in Supplementary Table 5, range from 0.1 to 0.2, suggesting a moderate model fit. This finding aligns with the results reported in Supplementary Table 4 based on the HL goodness-of-fit test. Potential reasons for the lack of model fit and possible approaches to address this issue in future research have been discussed above.

Supplementary Table 1. The mean p-values obtained from the Ljung-Box tests.

Symptom	p-value
Cardiac	0.408
Pulmonary	0.441
Sensation	0.517
Nausea	0.478
Movement	0.510
Pain	0.381
Memory	0.229
Fatigue	0.511
Anxiety	0.518
Depression	0.523

Supplementary Table 2. The average VIF values.

Symptom	VIF
Cardiac	1.867
Pulmonary	2.823
Sensation	2.221
Nausea	2.550
Movement	2.438

Pain	1.585
Memory	2.116
Fatigue	2.442
Anxiety	2.752
Depression	2.394

Supplementary Table 3. The average number of outliers.

Symptom	Number of Outliers
Cardiac	0.00
Pulmonary	0.00
Sensation	0.00
Nausea	1.11
Movement	0.00
Pain	0.00
Memory	0.00
Fatigue	0.00
Anxiety	0.00
Depression	0.00

Supplementary Table 4. The mean p-values obtained from the HL tests.

Symptom	p-value
Cardiac	1.41×10^{-3}
Pulmonary	3.90×10^{-4}
Sensation	3.56×10^{-3}
Nausea	1.08×10^{-3}
Movement	3.59×10^{-5}
Pain	9.78×10^{-4}
Memory	3.04×10^{-3}
Fatigue	3.33×10^{-4}
Anxiety	6.19×10^{-3}
Depression	5.12×10^{-4}

Supplementary Table 5. The average McFadden's R-squared values.

Symptom	Pseudo R-squared
Cardiac	0.2
Pulmonary	0.1
Sensation	0.1
Nausea	0.1
Movement	0.2
Pain	0.2
Memory	0.2
Fatigue	0.1
Anxiety	0.1
Depression	0.1

Supplementary Note II: Additional Simulations

Beyond the simulation studies outlined in the main manuscript, we conducted additional simulation experiments to showcase the performance of the proposed method in estimating personal temporal symptom networks across various scenarios. In the first scenario (a), we manipulated the structure of the generated temporal network by varying the rewiring probability $\theta \in \{0.0,0.3,0.5,0.8,1.0\}$. This transitioned the temporal network from a ring lattice ($\theta = 0.0$) to a small-world network ($0 < \theta < 1$) and finally to a random network ($\theta = 1.0$). In the second scenario (b), we manipulated the probability $p_B \in \{0.1,0.2,0.3,0.4,0.5\}$ of the Bernoulli distribution for the covariates $\boldsymbol{X}$, mirroring various prevalence rates observed in real-world situations. In the third scenario (c), we varied the number of nodes $n_{node} \in \{5,8,10,12,15\}$ considered in generating the temporal network. This variation represents different network sizes encountered in real data analysis. In the final scenario (d), we explored the impact of the strength of covariate-related correlation on estimating the personal temporal symptom network. This was achieved by setting different values for the covariate-related correlation between nodes, specifically $\gamma_{jj'l}^{1} \in \{1.0,1.5,2.0,2.5,3.0\}$. We performed the simulations at sample size $n = 1{,}000$. All other settings in these simulation scenarios were consistent with those described in the main manuscript's simulation section. Results of true positive rate (TPR), false positive rate (FPR), and Matthew's correlation coefficient (MCC) from these simulation experiments are presented in Supplementary Table 6. The results revealed that TPR values remained relatively stable when varying the rewiring probability θ, the Bernoulli probability p_B, the number of nodes n_{node}, and the strength $\gamma_{jj'l}^{1}$, except in the scenario when $p_B = 0.1$ and $\gamma_{jj'l}^{1} = 1.0$, which led to a large decrease in TPR. The FPRs are well controlled, except for scenarios with small values of n_{node} (eg, 5 or 8), while the MCC values are quite stable across different scenarios.

Supplementary Table 6. The TPR, FPR, and MCC values for the identification of coefficients in the simulated personal temporal symptom networks. Scenario (a): varying rewiring probability θ; (b) varying the Bernoulli probability for covariates $\boldsymbol{X}$; (c) varying the number of nodes; (d) varying the strength of the covariate-related correlation.

Scenario		TPR	FPR	MCC
a	$\theta = 0.0$	0.994 (0.018)	0.021 (0.007)	0.781 (0.049)
	$\theta = 0.3$	0.977 (0.048)	0.017 (0.006)	0.799 (0.050)
	$\theta = 0.5$	0.950 (0.086)	0.016 (0.006)	0.794 (0.056)
	$\theta = 0.8$	0.958 (0.081)	0.016 (0.006)	0.797 (0.055)
	$\theta = 1.0$	0.965 (0.075)	0.016 (0.006)	0.805 (0.057)
b	$p_B = 0.1$	0.886 (0.066)	0.010 (0.004)	0.813 (0.054)
	$p_B = 0.2$	0.951 (0.053)	0.013 (0.005)	0.819 (0.051)
	$p_B = 0.3$	0.975 (0.042)	0.015 (0.005)	0.815 (0.046)
	$p_B = 0.4$	0.979 (0.038)	0.016 (0.006)	0.808 (0.047)
	$p_B = 0.5$	0.977 (0.048)	0.017 (0.006)	0.799 (0.050)
c	$n_{node} = 5$	0.980 (0.031)	0.052 (0.018)	0.831 (0.051)
	$n_{node} = 8$	0.986 (0.031)	0.028 (0.009)	0.797 (0.050)
	$n_{node} = 10$	0.977 (0.048)	0.017 (0.006)	0.799 (0.050)
	$n_{node} = 12$	0.975 (0.056)	0.011 (0.004)	0.813 (0.050)
	$n_{node} = 15$	0.953 (0.078)	0.006 (0.002)	0.826 (0.054)
d	$\gamma_{jj'l} = 1.0$	0.820 (0.072)	0.011 (0.005)	0.759 (0.058)
	$\gamma_{jj'l} = 1.5$	0.926 (0.070)	0.014 (0.006)	0.794 (0.052)
	$\gamma_{jj'l} = 2.0$	0.952 (0.060)	0.015 (0.005)	0.803 (0.051)
	$\gamma_{jj'l} = 2.5$	0.965 (0.058)	0.016 (0.006)	0.802 (0.051)
	$\gamma_{jj'l} = 3.0$	0.973 (0.048)	0.016 (0.006)	0.804 (0.049)

Supplementary Figure 1. Tests of differences between the identified pairwise associations in the constructed personal temporal symptom network based on the adult survivors of childhood cancer from SJLIFE study (n=2,000).

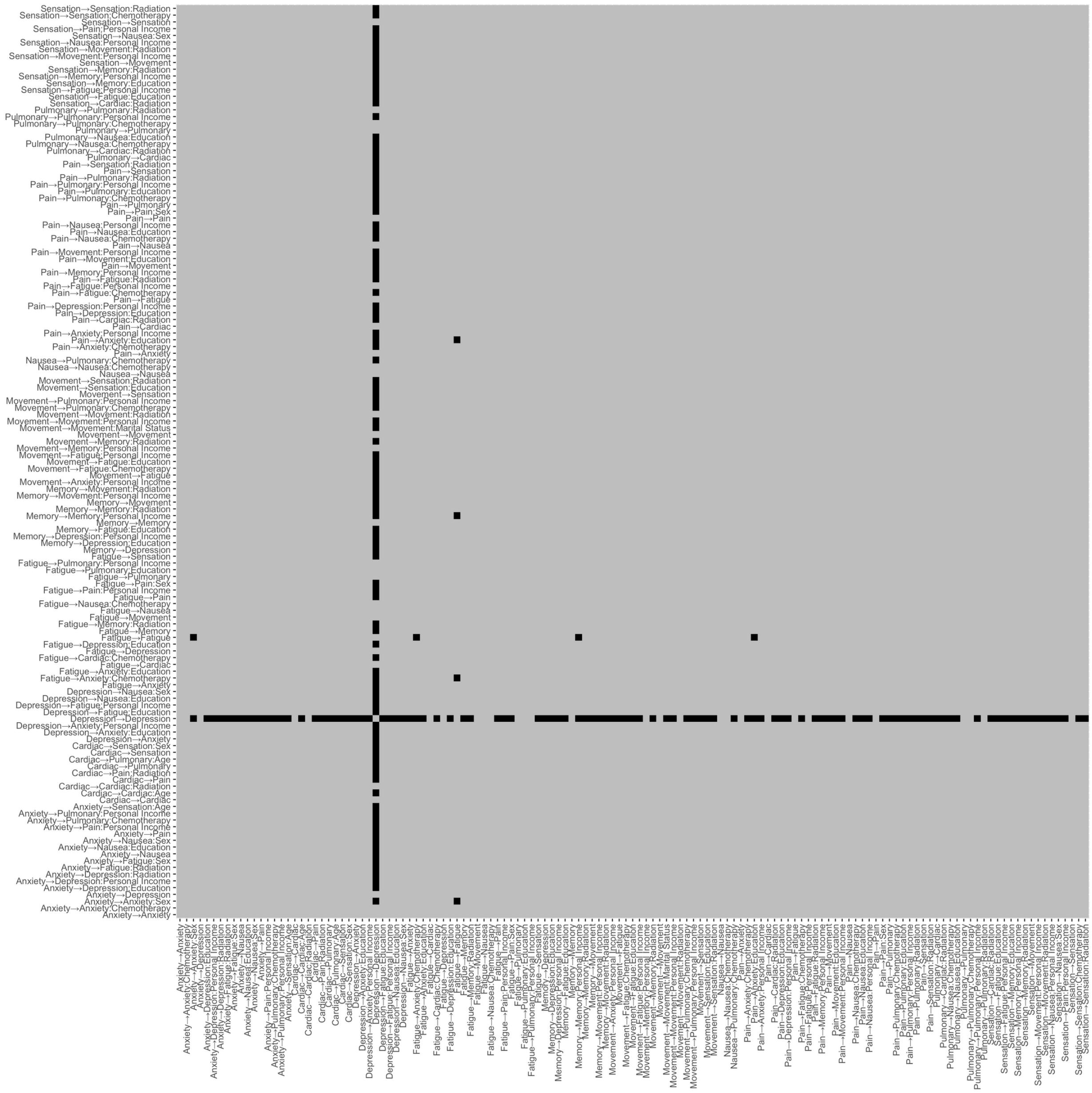

Black tiles indicate statistically significant differences in pairwise symptom associations, while grey tiles indicate non-significant differences.

Supplementary Figure 2. Tests of differences between the out-strength centrality for symptoms in the constructed personal temporal symptom network for the second representative subject.

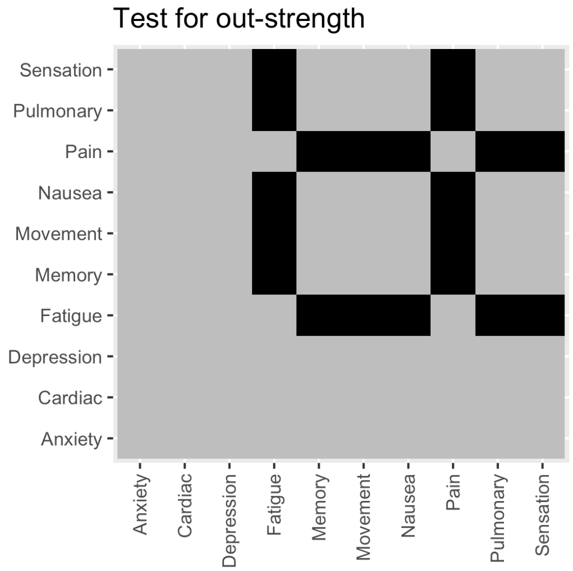

The representative cancer survivor has more risk factors: she is a 39.9-year-old female at survey with below college/post-graduate education, earning <$20,000 annually, who received chemotherapy and/or radiation. Black tiles indicate statistically significant differences in pairwise symptom associations, while grey tiles indicate non-significant differences.
