# Supplementary material for: Estimating personal temporal symptom networks for childhood cancer survivors
Source: Commun Med (Lond). 2025 Aug 29;5:377. doi: 10.1038/s43856-025-01105-0 (PMC12394638; doi:10.1038/s43856-025-01105-0)
Supplement: Supplementary file 3 — Description of Additional Supplementary files [file 43856_2025_1105_MOESM3_ESM.pdf]

Description of Additional Supplementary files

File name: Supplementary Data

Description: The source data for Figures 4-7 and Supplementary Figures 1-2.
